# Effects of chromosomal translocation characteristics on fertilization and blastocyst development — a retrospective cohort study

**DOI:** 10.1186/s12920-023-01715-4

**Published:** 2023-11-01

**Authors:** Shanshan Wu, Jianrui Zhang, Yichun Guan, Bingnan Ren, Yuchao Zhang, Xinmi Liu, Kexin Wang, Mingmei Zhang, Zhen Li

**Affiliations:** Reproductive Medicine Center, Third Affiliated Hospital of Zhengzhou University, Zhengzhou, China

**Keywords:** Preimplantation genetic testing for chromosomal structural rearrangements, Reciprocal translocation, Robertsonian translocation, Fertilization, Blastocyst development

## Abstract

**Objective:**

To determine the effect of different translocation characteristics on fertilization rate and blastocyst development in chromosomal translocation patients.

**Methods:**

This retrospective cohort study was conducted at the Third Affiliated Hospital of Zhengzhou University From January 2017 to December 2022.All couples were diagnosed as reciprocal translocation or Robertsonian translocation by karyotype of peripheral blood lymphocytes test. After adjusting for confounding factors, the effect of chromosomal rearrangement characteristics, such as carrier sex, translocation type, chromosome length and break sites, on fertilization rate and embryo development were analysed separately using multiple linear regression.

**Results:**

In cases of Robertsonian translocation (RobT), the carrier sex plays an independent role in fertilization rate, and the male carriers was lower than that of female carriers (76.16% vs.86.26%, *P* = 0.009). In reciprocal translocation (RecT), the carrier sex, chromosome types and break sites had no influence on fertilization rate, blastocyst formation rate (*P* > 0.05). However, patients with human longer chromosomal (chromosomes 1–5) translocation have a lower available blastocyst formation rate (Group AB vs. Group CD: 41.49%vs.46.01%, *P* = 0.027). For male carriers, the translocation types was an independent factor affecting the fertilization rate, and the RobT was the negative one (*B* = − 0.075, *P* = 0 0.009). In female carriers, we did not observe this difference (*P* = 0.227).

**Conclusions:**

In patients with chromosomal translocation, the fertilization rate may be influenced by carrier sex and translocation type, chromosomes 1–5 translocation may adversely affect the formation of available blastocysts. Break sites have no role in fertilization and blastocyst development.

**Supplementary Information:**

The online version contains supplementary material available at 10.1186/s12920-023-01715-4.

## Introduction

Chromosomal balanced translocation(BT) encompass reciprocal translocation (RecT) and Robertsonian translocation (RobT), with a prevalence of 0.2% and 0.1% in the population, respectively [1, 2]. Those patients usually behave normally, but they are prone to abnormal gamete production, leading to fertility problems [3, 4]. Preimplantation genetic testing for structural rearrangements (PGT-SR) by second-generation sequencing (NGS) may improve the pregnancy outcomes in patients [5].This technique is extensively employed in clinical settings [6]. Research has indicated that individuals carrying chromosomal translocations exhibit a reduced rate of blastocyst formation and delayed blastocyst development. Various translocation characteristics, including carrier sex, translocation types and chromosome length, may exert distinct detrimental effects on the process of blastocyst development, as evidenced by relevant literature [7–9]. Scholars generally believe that the carrier sex and translocation type have significant effects on chromosome ploidy of blastocysts [10–12]. There is no inevitable relationship between breakpoint positions and blastocyst outcomes [9]. Some researchers suggested that male carriers may be a risk factor for fertilization rate [13], this may be related to differences in the mechanism of gamete meiosis and the probability of errors between male and female gametes [14]. So far, the impact of carrier sex on fertilization rates remains a subject of considerable debate [13, 15, 16].

Previous research has established that patients with chromosomal polymorphisms exhibit a notably lower fertilization rate compared to those with healthy chromosomes [17]. Furthermore, it has been observed that the negative impact on fertilization rate is more pronounced when the carrier is male rather than female [18, 19]. Based on previous research, we know that there is a significant correlation between carrier sex, translocation type and blastocyst outcome [20]. However, the existing body of research on the influence of different translocation characteristics on fertilization and blastocyst development remains insufficient [13, 16]. PGT-SR may improve pregnancy outcome in these patients, but this would be futile in the absence of a fertilized oocyte and an available blastocyst.

To address the inadequacies of the current studies, we comprehensively analysed the potential association of translocation characteristics (such as chromosome length, breakpoint, translocation type and carrier sex) with fertilization rate, blastocyst formation rate and available blastocyst rate. And to reduce the potential bias of duplicate data, we only included the first PGT-SR cycle of each couple. The categorization of our study sample was informed by previous literature [9, 15, 21]. Specifically, the population was divided into two groups according to whether the acrocentric chromosomes were translocated [15]; they are divided into three groups based on the breakpoint (pp/pq/qq) [9]. The division of chromosome length is based on the *International System for Human Cytogenetic Nomenclature(ISCN 2020)*, which divides 46 human chromosomes into longer(group A/B), medium(group C/D), and shorter chromosomes(group E/F/G) [22] .

## Materials and methods

### Study population

This study is a retrospective cohort analysis that examines the clinical data of 414 patients who underwent preimplantation genetic testing for chromosomal structural rearrangements (PGT-SR) at the Reproductive Medicine Center of the Third Affiliated Hospital of Zhengzhou University from January 1, 2017 to December 31, 2022. All participating couples underwent confirmation of their genetic makeup through peripheral blood lymphocyte karyotyping, revealing that one partner from each couple possessed a balanced chromosomal translocation. The study excluded couples with complex chromosome rearrangements or couples in which both partners had chromosomal abnormalities. And we only included data from each patient’s initial PGT-SR cycle. The study was approved by the Ethics Committee of the Third Affiliated Hospital of Zhengzhou University, which also waived for informed consent. The approval number was 2022-414-01, and the date approval occurred was 28 November 2022.

### Grouping

As described in the *ISCN (2020)* [22], the human chromosomes are divided into seven groups (denoted A–G) according to the chromosome length and position of the centromere. Groups A and B consist of large human chromosomes. Groups C and D consist of medium-size chromosomes. Groups E, F and G are human small chromosomes. The subgroups of this study are as follows, in the RecT, group AB means that at least one of the translocated chromosomes belongs to the large chromosomes of the karyotype (AA + AB + AC + AD + AE + AG + BB + BC + BD + BE + BF + BG); group CD means that one of the chromosomes belongs to the medium-size chromosomes (CC + CD + CE + CF + CG + DD + DE + DF); group EFG means that one of the chromosomes belongs to the small chromosomes(EF + EG + FG). Due to the peculiarity of the RobT, they were divided into DD (both translocated chromosomes are belong to medium chromosomes), DG (one belongs to the medium chromosomes and the other belongs to the small chromosomes) and GG (both chromosomes are belong to the small chromosomes) groups. According to the chromosome break sites, the cohort was divided into 3 groups, as follows: pq (one breakpoint was located on the long arm of the chromosome, while the other was located on the short arm), pp (both breakpoints were located on the short arm), qq (both breakpoints were located on the long arm). In our data, translocation without the acrocentric chromosome (Acr-ch) is called non Acr-ch group; otherwise, they are called Acr-ch group. The above groupings are based on the *ISCN(2020)* and previous literature [22, 23].

### Semen collection and handling

The individual practices sexual abstinence for a period of 3–7 days in order to obtain semen by masturbation. The volume, vitality, and concentration of the semen are then recorded according to the guidelines of the WHO Manual for Human Sperm Testing. In the case of patients undergoing percutaneous epididymal sperm aspiration (PESA) or testicular sperm aspiration (TESA), the procedure involves the administration of lidocaine anesthesia, followed by the collection of epididymal fluid or testicular tissue by surgical puncture. The tissue is then completely disrupted using a 1 ml syringe needle to maximize the release of sperm into the culture medium. The collected semen or tissue suspension was washed by centrifugation, and the treated semen was examined under a microscope and centrifuged for later use.

### Ovulation-inducing, insemination and embryo culture

All patients underwent ultrasound scan and a serum sex hormone evaluation on the third day of the menstrual cycle to assess ovarian reserve function. Ovarian stimulation protocols include gonadotropin-releasing hormone (GnRH) antagonist, GnRH agonist, or progestin-primed ovarian stimulation (PPOS) [24]. The growth of follicles was monitored during ovulation, and when at least two follicles had reached 18 mm in diameter or the dominant follicle was ≥ 16 mm in diameter, human chorionic gonadotropin or GnRHa was injected as a trigger. Oocytes were retrieved under vaginal ultrasound guidance after a period of 36 h. The oocyte corona cumulus complex (OCCC) was observed through microscopic examination, and the number of oocytes retrieved was documented. The OCCC was subsequently cultivated for a duration of 2 h, during which the granulosa cells were eliminated. Following this, mature oocytes (MII) were subjected to fertilization via intracytoplasmic sperm injection (ICSI). Embryos were cultured from pronuclear stage to cleavage stage in G1™ Plus (Vitrolife) after fertilization and then from cleavage stage to blastocyst stage in G-2™ Plus (Vitrolife) [25]. Embryo quality was evaluated based on previous literature [26], and day 3 embryos with a score of grade III or higher were designated as D3 available embryos in our institution. In accordance with the Vienna Consensus [27], the available D3 embryos rate = no. of D3 available embryos/no. of normally fertilized oocytes. The D5/D6/D7 blastocysts were observed and evaluated based on Gardner’s scoring system [28]. At our institution, blastocysts with a score higher than 3BC were defined as available blastocysts, and selected for biopsy.

The fertilization rate = number of 2PN and 2BN/number of MII oocytes × 100%. Blastocyst formation rate = number of blastocysts/number of blastocysts cultured × 100%. Available blastocyst formation rate = available blastocyst/ number of blastocysts cultured × 100%.

### Statistical analysis

The statistical analysis was performed with SPSS 25.0. Graphs were generated using GraphPad Prism 8. The data are described as the mean ± standard deviation, median (interquartile range) [M(Q1, Q3)], or percentage (%). The t-test was used to compare the numeric variables, and the chi-square test was used to compare the categorical variables. Variables that were significant in the univariate analysis were included in the multivariate analysis. After excluding confounding factors, the effect of chromosome structural rearrangement characteristics on fertilization rate, blastocyst formation rate and available blastocyst formation rate was analysed separately using multiple linear regression. And we included both female and male age variables in the multivariate analysis. Variables that are significant sessed for covariance before inclusion in the multiple regression equation. The variance inflation factor for each variable was less than 5; thus, the variables were considered to have no covariance. *P* value < 0.05 was considered statistically significant.

## Results

**Fig. 1:**
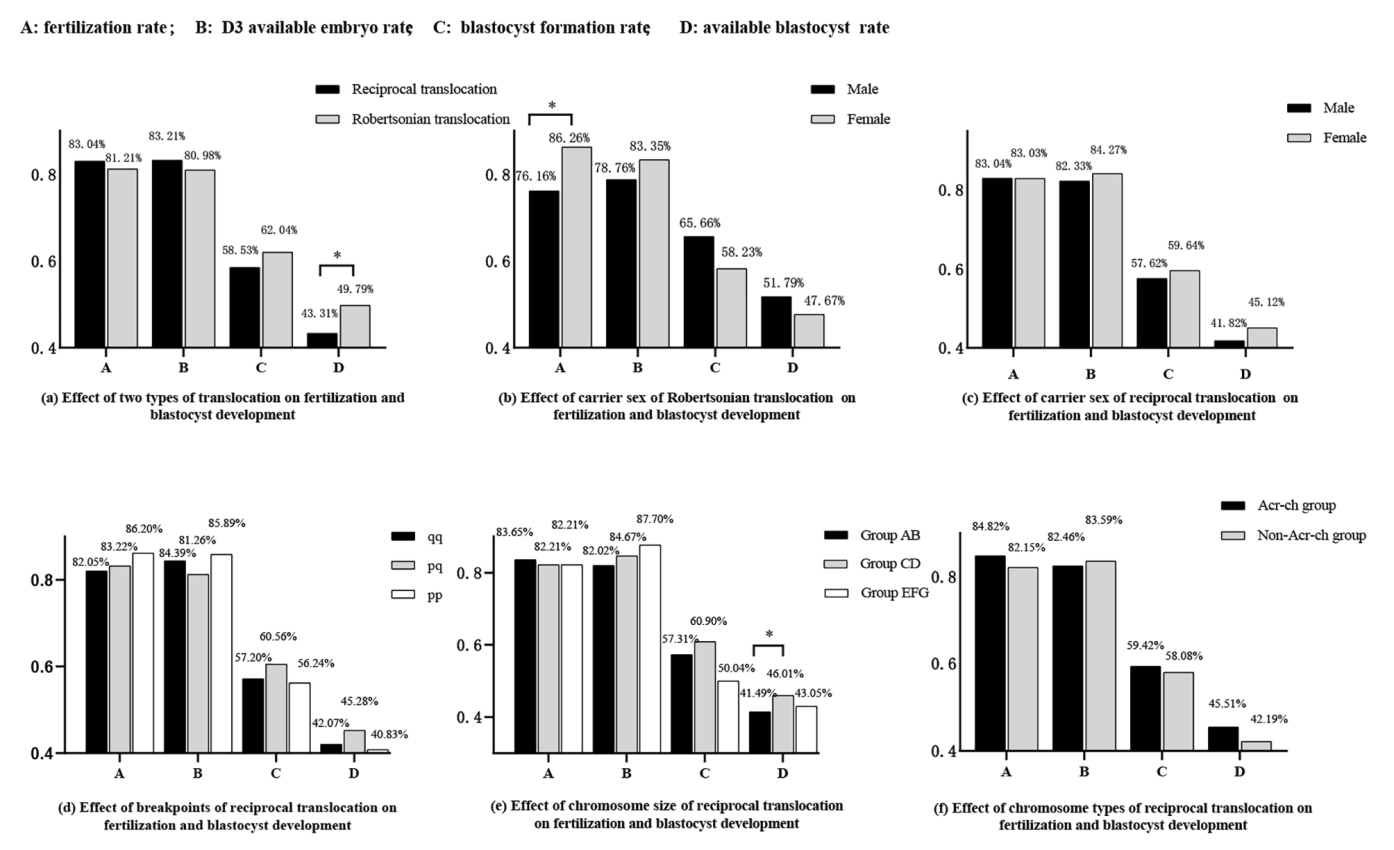
Effect of translocation characteristics on fertilization and blastocyst development. **(a)** *t-test is used to obtain the p value, *P* < 0.05. **(b)(e)** *multivariate regression is used to obtain the p value, *P* < 0.05. **(d)** pq represents one break site in chromosome long arm, another in the short arm;pp represents 2 break sites are in the chromosome short arms; qq represents 2 break sites are in the chromosome long arms. **(e)** group AB means that at least one of the translocated chromosomes belongs to the large chromosomes; group CD means that one of the chromosomes belongs to the medium-size chromosomes; group EFG means that one of the chromosomes belongs to the small chromosomes. **(f)** Acr-ch represents Acrocentric chromosome

A total of 414 PGT cycles were included in this study, including 299 cycles of RecT (female carrier: 135 cycles; male carrier: 164 cycles) and 115 cycles of RobT (female carrier: 56 cycles; male carrier: 59 cycles). Basic information for all study populations is presented in Table 1. Information on fertilization and blastocyst development for different translocation characteristics is shown in Fig. 1.

### Main outcomes of the RecT patients

The average fertilization rate was 83.04% in the RecT. And for the AB, CD and EFG groups were 83.65%, 82.21% and 82.21%, the qq, pq and pp groups were 82.05%, 83.22% and 86.20%, respectively. The blastocyst formation rates of the AB, CD and EFG groups were 57.31%, 60.92% and 50.04% respectively, the available blastocyst rates were 41.49%, 46.01% and 43.05%. The blastocyst formation rates of the qq, pq and pp groups were 57.20%, 60.56% and 56.24%, the available blastocyst rates were 42.07%, 45.28% and 40.83%, respectively. The blastocyst formation rates of the male and female groups were 57.62% and 59.64%, respectively, and the available blastocyst rates were 41.82% and 45.12%. The significant variables (P < 0.05) in the univariate analysis results were included in the multivariate analysis. Currently, it is generally accepted that age has an unavoidable impact on the outcome, so we included both male and female age in the multivariate analysis. The effects of carrier sex, chromosome length, breakpoints and chromosome type on fertilization rate, blastocyst formation rate and available blastocyst rate were analyzed separately using multiple linear regression. The results showed that the carrier sex, breakpoints and chromosome type did not affect fertilization and blastocyst development (*P* > 0.05) (Table 2). The available blastocyst rate of the AB group (chromosomes 1–5) was significantly lower (*B*=-0.060, *P* = 0.027). The results of univariate linear regression analysis are shown in Supplemental Table 1.

### Main outcomes of the RobT patients

The average fertilization rate was 81.21% in the RobT(female RobT 86.26%, male RobT 76.16%). The DD, DG and GG groups were 80.55%, 84.15% and 67.11% respectively. The blastocyst formation rates of the DD, DG and GG groups were 62.97%, 62.60% and 15.63%, respectively, the available blastocyst rates were 49.74%, 52.48% and 12.50%. Multivariable analysis was used to explore the effects of carrier sex and chromosome length on fertilization rate, blastocyst formation rate and available blastocyst rate respectively. Due to the small sample size of the GG group (only 2 cycles), they were not included. The results showed that carrier sex was an independent influencing factor, and the fertilization rate was significantly lower in male carriers than in female carriers (*B* =-0.091, *P* = 0.009) (Table 3). The results of univariate linear regression analysis are shown in Supplemental Table 2.

### Main outcomes of the different translocation types

The cohort was divided into two groups based on the carrier sex, the results of multivariable analysis showed that in male carriers, the type of translocation significantly affects fertilization rate (*B* =-0.075, *P* = 0.009) and available blastocyst rate (*B* = 0.087, *P* = 0.012). In female carriers, the type of translocation had no effect on the fertilization rate (*P* = 0.227) (Table 4). The results of univariate analysis are shown in Supplemental Tables 3, 4.

**Table 1:** Basic information description of patients [$\bar x$ ± s, M(Q1, Q3), %]

Item			Reciprocal translocation	Robertsonian translocation	*P* value
No. of cycles			299	115	
Female age (years)			30.09 ± 4.61	30.08 ± 3.75	0.986
Male age (years)			30.93 ± 5.10	30.83 ± 3.74	0.830
Female BMI (kg/m^2^)			23.85 ± 3.16	23.94 ± 3.20	0.812
Gn dose/1000 (U)			2.79 ± 0.91	2.79 ± 0.88	0.894
Gn days			11.0 ± 2.1	11.0 ± 2.1	0.874
AMH(pmol/L)			30.09 ± 20.63	28.78 ± 18.07	0.550
Basal FSH (U/L)			6.45(5.40, 7.45)	6.52(5.55, 7.94)	0.303
Types of infertility		primary	38.13%(114/299)	40.87%(47/115)	0.608
		secondary	61.87%(185/299)	59.13%(68/115)	
Ovarian stimulation		GnRH agonist	42.47%(127/299)	42.61%(49/115)	0.665
		Antagonists	47.83%(143/299)	50.43%(58/115)	
		PPOS	9.70%(29/299)	6.96%(8/115)	
Semen volume			1.94 ± 0.27	1.90 ± 0.32	0.296
Semen motility			37.58 ± 12.98	33.07 ± 14.45	0.002
Fertilization rate (2PN)			0.83 ± 0.17	0.81 ± 0.19	0.356
D3 available embryos rate			0.83 ± 0.18	0.81 ± 0.22	0.308
Blastocyst formation rate			0.58 ± 0.25	0.62 ± 0.23	0.200
Available blastocyst formation rate			0.43 ± 0.23	0.49 ± 0.22	0.010
*Different characteristics of Translation*					
Break site	pq		129(43.1%)	0	NE
	qq		135(45.2%)	100%	
	pp		35(11.7%)	0	
Carrier sex	Male		164(54.8%)	59(51.3%)	0.517
	Female		135(45.2%)	56(48.7%)	
Chromosome length	AB group		173(57.9%)	0	NE
	CD group		118(39.5%)	0	
	EFG group		8(2.7%)	0	
	DD group		0	84(73.0%)	
	DG group		0	29(25.2%)	
	GG group		0	2(1.7%)	
Chromosome types	Acr-ch		100(33.4%)	100%	NE
	Non Acr-ch		199(66.6%)	0	

Notes: BMI represents body mass index; AMH represents anti-Müllerian hormone; FSH represents follicle-stimulating hormone; Gn represents gonadotropins; pq represents a break site in chromosome long arm, another in chromosome short arm; pp represents 2 break sites are in chromosome short arms; qq represents 2 break sites are in chromosome long arms; Acr-ch represents Acrocentric chromosome

**Table 2:** Multivariate linear regression analysis of the impact of translocation characteristics on fertilization and blastocyst development in RecT patients

Item		fertilization rate ^a^		blastocyst formation rate ^b^		available blastocyst rate ^c^	
		*B(95.0%CI)*	*P*	*B(95.0%CI)*	*P*	*B(95.0%CI)*	*P*
Carrier sex	Male	0.005(-0.035,0.045)	0.807	-0.015(-0.072,0.043)	0.614	-0.025(-0.076,0.027)	0.349
	Female	0		0		0	
Chromosome type	Acr-ch	0.033(-0.009,0.075)	0.128	0.020(-0.041,0.081)	0.522	0.044(-0.010,0.099)	0.106
	Non Acr-ch	0		0		0	
Chromosome length	Group AB	0.006(-0.035,0.048)	0.767	-0.045(-0.104,0.015)	0.140	-0.060(-0.112, -0.007)	0.027
	Group EFG	< 0.001(-0.125,0.125	0.997	-0.111(-0.291,0.068)	0.223	-0.029(-0.189,0.130)	0.720
	Group CD	0		0		0	
Break sites	pp group	0.026(-0.040,0.092)	0.443	-0.011(-0.105,0.084)	0.825	-0.024(-0.109,0.061)	0.579
	pq group	0.006(-0.036,0.049)	0.767	0.027(-0.034,0.088)	0.387	0.025(-0.029,0.080)	0.359
	qq group	0		0		0	

Notes: Acr-ch represents Acrocentric chromosome; pq represents a break site in chromosome long arm, another in chromosome short arm;pp represents 2 break sites are in chromosome short arms; qq represents 2 break sites are in chromosome long arms;

“a”: adjusting for confounding factors: female age, male age, Gn dose, Days of Gn;

“b”: adjusting for confounding factors: female age, male age, Gn dose, basal FSH;

“c”: adjusting for confounding factors: female age, male age, Gn dose, Days of Gn, basal FSH

**Table 3:** Multivariate linear regression analysis of the impact of translocation characteristics on fertilization and blastocyst development in RobT patients

Item			fertilization rate ^a^		blastocyst formation rate ^b^		available blastocyst rate ^c^	
			*B(95.0%CI)*	*P*	*B(95.0%CI)*	*P*	*B(95.0%CI)*	*P*
Carrier sex	Male	-0.093(-0.162, -0.024)		0.009	0.068(-0.017,0.153)	0.115	0.042(-0.040,0.124)	0.313
	Female	0			0		0	
Chromosome length	DD Group	-0.039(-0.121,0.042)		0.343	0.005(-0.092,0.101)	0.925	-0.034(-0.127,0.059)	0.469
	DG Group	0			0		0	

Notes: DD group represents: both translocated chromosomes are belong to medium chromosomes; DG group represents: one belongs to the medium chromosomes and the other belongs to the small chromosomes

“a”: adjusting for confounding factors: female age, male age, Gn dose, AMH;

“b”: adjusting for confounding factors: female age, male age, Gn dose, basal FSH;

“c”: adjusting for confounding factors: female age, male age, Gn dose, Days of Gn, basal FSH

**Table 4:** Multivariate analysis of the effects of translocation types on fertilization and blastocyst development

Item		fertilization rate ^a^		blastocyst formation rate ^b^		available blastocyst rate ^c^	
		*B(95%CI)*	*P*	*B(95%CI)*	*P*	*B(95%CI)*	*P*
*Male carriers*							
Types	RobT	-0.075(-0.131,-0.019)	0.009	0.072(-0.003,0.148)	0.061	0.087(0.019, 0.155)	0.012
	RecT	0		0		0	
*Female carriers*							
Types	RobT	0.032(-0.020,0.083)	0.227	-0.015(-0.092,0.061)	0.692	0.024(-0.047,0.096)	0.501
	RecT	0		0		0	

Notes: RecT represents reciprocal translocation; RobT represents Robertsonian translocation;

“a”: adjusting for confounding factors: female age, male age, Gn dose, days of Gn, basal LH, AMH;

“b”, “c”: adjusting for confounding factors: female age, male age, Gn dose, basal FSH

## Discussion

This study, for the first time, systemically compared the effects of the characteristics of patients with chromosomal structural rearrangements on fertilization and blastocyst development. Adjusting for potential confounders using multivariate linear regression analysis. Our data showed that translocation types and carrier sex may potentially exert an influence on fertilization rate, while break sites and chromosome types do not play a significant role. Longer chromosome translocations (chromosomes 1–5) may be a risk factor for obtaining available blastocysts. And translocation types may have an impact on the formation of available blastocysts.

### Effect of translocation types on fertilization and blastocyst development

Our data shows that, in the male group, the fertilization rate of the RobT was lower than that of the RecT (*B* =-0.069, *P* = 0.027). This finding is consistent with the observations of *Findikli* et al. who also reported a lower fertilization rate for the RobT compared to the RecT (58.8% vs. 64.5%) [29]. We speculate that this may be because acrocentric chromosomes (Acr-ch, RobT’s chromosomes) are unstable during meiosis and mitosis, resulting in abnormal distribution of genetic material in the gametes. This hypothesis is supported by previous research findings. By analyzing the segregation patterns of the two translocation types, *Zhang* et al. suggested that the involvement of Acr-ch hinders spermatogenesis [30, 31]. *Silvia Garagna* et al. linked Robertsonian chromosomes to subfertility in mice, positing that the presence of Robertsonian chromosomes leads to impaired spermatogenesis [32]. Using transmission electron microscopy analysis of spermatozoa from 45, XY, der (14; 22) patients (the RobT), *Baccio* et al. found that the sperm had abnormal ultrastructural defects associated with immaturity [33]. Additionally, the gametes of these two translocation types form distinct unique chromosome structures during meiosis (RecT forms tetravalents, while RobT forms trivalents) [31, 34]. The diminished rate of fertilization observed in RobT patients may be attributed to the inherent instability of the trivalent structure, which consequently increases the likelihood of generating unbalanced gametes. On the other hand, the unbalanced sperm of patients with RobT exhibit increased susceptibility to exogenous fragmentation factors, which have the potential to disrupt the genetic material of the sperm [35, 36], and impede the regular insemination process.

As we know, blastocyst culture is the process of further screening of embryos. The analysis of our data reveals that there is no significant difference in the fertilization rate and D3 availability embryo rate between the two types of translocation. However, it is noteworthy that the available blastocyst rate of the RecT is lower compared to the RobT. Additionally, the trend in blastocyst formation rate follows a similar pattern, although statistical significance is not observed, as illustrated in Fig. 1(a). Therefore, we speculate that the screening of embryos primarily occurs during the blastocyst formation stage, which is consistent with the results observed with time-lapse imaging and embryo genetic testing [37, 38]. *Mateu-Brull* et al. conducted a comparative analysis of embryo biopsy outcomes on day 3 and day 5/6, revealing a higher proportion of normal embryos at the blastocyst stage compared to the cleavage stage [23]. Our suggestion is consistent with the prevailing opinion that the blastocyst outcome of the RecT is inferior to that of the RobT [20, 29]. This phenomenon could potentially be attributed to the diminished developmental capacity of embryos and the increased likelihood of chromosomally abnormal embryos in patients with RecT [23].

### Effect of carrier sex on fertilization and blastocyst development

We know that female and male gametes develop differently, as female germ cells develop to metaphase I before birth and arrest at this stage [39], while male sperm develop continuously from puberty [40]. Spermatogonia undergo repeated mitoses before the onset of meiosis, which increases the chance of chromosome segregation errors. And the men with chromosomal structural abnormalities have significantly higher rates of sperm DNA fragmentation [35], which affects the fertilization rate [41]. The physical observation we normally use cannot accurately identify these sperm [42], so ICSI insemination is likely to lead to the selection of sperm with abnormal karyotypes, which can result in lower fertilization rates. Notably, DNA breaks are only associated with structural chromosomal abnormalities [43].According to Academician *Chen Zijiang*, the cause of failed fertilization is not simply the result of the sperm not entering the follicle; rather, it may be the result of various causes of oocyte plasma inactivation or abnormal sperm chromosome depolymerization. For example, male *PLCZ1* gene mutation leads to oocyte activation disorder [44]. A previous study suggested that chromosomal polymorphisms negatively affect the fertilization rate [17],and male carriers have significantly lower fertilization rates [18]. *Zhanhui* et al. concluded that male carrier is thought to be a risk factor [19].

Our data showed that, in the RobT, male carrier was identified as a negative factor influencing the fertilization rate. A previous study [45] indicated that the male carriers had a lower fertilization rate than female (58% vs. 67.9%), but the difference was not statistically significant, possibly due to the small sample size (only 66 cycles). Data from a recent study showed that [16], in cases of RobT, the fertilization rate of male carriers was significantly lower than that of females (61.04% vs. 65.70%, *P* < 0.001), but this phenomenon was not present in the RecT, which supports the results of our study. Some researchers had a different view, *Findikli* et al. concluded that the fertilization rate of male RecT patients was lower than that of female (the sample size was only 24 cycles) [29]. *Li* et al. believed that female RecT patients had a lower fertilization rate than males (78.8% vs. 83.8%, *P* < 0.05), [13] but the difference was not observed in the RobT (79.4% vs. 85.1%, *P* > 0.05). The observations of these two studies [13, 29] were contradictory, which may be due to differences between research centers and the influence of potential confounders. *Stahl, A* et al. indicated that in oocytes, the Acr-ch are distributed across multiple regions, while in spermatogonia, they are localized in a single region [46]. This distributional behavior may explain the differences between male and female RobT. As we know, checkpoint mechanisms exist in human cells. When the chromosomes are abnormal, this mechanism is triggered, leading to meiotic arrest and reducing the production of abnormal gametes. However, this checkpoint mechanism is not fully effective in eliminating all abnormal cells [47]. Interestingly, RobT mouse models have shown that this checkpoint mechanism has low stringency in male mice rather than female [48]. However, there is insufficient evidence to determine whether this phenomenon exists in humans, although differences in the mechanisms of gamete meiosis and the probability of errors between males and females have been noted [14]. Up to now, few studies have been able to comprehensively dissect the mechanisms underlying this phenomenon, therefore, further expansion of the data and more rigorous basic research are needed to verify this conclusion in the future. Consistent with previous studies, we did not observe this sex effect in the RecT [15].

This study suggests that there is no significant difference in the blastocyst formation rate and available blastocyst rate between male and female carriers. We believe that the sex differences caused by the above mechanisms have little or no effect on blastocyst development after fertilization. Currently, the effect of carrier sex on blastocyst development is controversial [8, 49]. It may be due to the bias of population characteristics and sample size in different research centers, which leads to the differences of these research results. In the future, multi-center and large sample size studies are needed.

### Effects of chromosome size and breakpoint on fertilization and blastocyst development

In recent years, the impact of chromosome length and breakpoints on normal embryos has gradually attracted scholars’ attention [9, 23], but there is a dearth of reliable evidence in this area. To address the inadequacies of the current studies, we have conducted a preliminary investigation. It is worth noting that our data suggest that human longer chromosomal translocation (Chromosomes 1–5) is a risk factor for obtaining available blastocysts. It may be related to the poor developmental potential of human longer chromosomal translocation embryos. Previous research supports our conclusion that the longer the translocated chromosome, the more difficult it is to form a blastocyst [50]. A review of the previous literature indicated that human long chromosomes (chromosomes 1–5) exhibit a higher susceptibility to chromosome segregation errors compared to shorter chromosomes, potentially due to their elevated abundance of adhesion proteins [51]. Furthermore, it has been proposed that the majority of abnormal chromosomes found in aneuploid embryos are human long chromosomes (chromosomes 1, 2, 4, and 9) [52]. In contrast to previous study, only the initial cycle of each couple was included in this study to avoid the confounding effect of repeated measurement data from multiple cycles of a couple [24].

The limitation of this study is that these results come from our limited sample size, and in the future, we will conduct a multicenter study with a large sample size. And it is unsuitable to apply these findings to couples with complex chromosome rearrangements as they were excluded from the study.

In conclusion, translocation types and carrier sex may potentially exert an influence on fertilization rate. And human longer chromosome translocation (chromosomes 1–5) may be a risk factor for obtaining available blastocysts. And translocation types may have an impact on the formation of available blastocysts. The break sites play no role in fertilization and blastocyst development. Our limited data can provide some references for researchers and accurate genetic counseling.

### Electronic supplementary material

Below is the link to the electronic supplementary material.

Supplementary Material 1

## Data Availability

The datasets used during the current study are available from the corresponding author on reasonable request.

## References

[1] Therman E, Susman B, Denniston C (1989). The nonrandom participation of human acrocentric chromosomes in Robertsonian translocations. Ann Hum Genet.

[2] Jacobs P, Browne C, Gregson N, Joyce C, White H (1992). Estimates of the frequency of chromosome abnormalities detectable in unselected newborns using moderate levels of banding. J Med Genet.

[3] Wan X, Li L, Liu Z, Fan Z, Yu L (2021). Recurrent spontaneous abortion related to balanced translocation of chromosomes: two case reports. J Med Case Rep.

[4] Iews M, Tan J, Taskin O, Alfaraj S, AbdelHafez F, Abdellah A (2018). Does preimplantation genetic diagnosis improve reproductive outcome in couples with recurrent pregnancy loss owing to structural chromosomal rearrangement? A systematic review. Reprod Biomed Online.

[5] Ma X, Xu X, Mao B, Liu H, Li H, Liu K (2021). Chromosomal analysis for embryos from balanced chromosomal rearrangement carriers using next generation sequencing. Mol Reprod Dev.

[6] Fesahat F, Montazeri F, Hoseini SM. Preimplantation genetic testing in assisted reproduction technology. J Gynecol Obstet Hum Reprod. 2020;49(5). 10.1016/j.jogoh.2020.101723 32113002

[7] Liu Y, Shen J, Yang R, Zhang Y, Jia L, Guan Y (2022). The relationship between human embryo parameters and De Novo Chromosomal Abnormalities in preimplantation genetic testing cycles. Int J Endocrinol.

[8] Song H, Shi H, Yang E, Bu Z, Jin Z, Huo M (2021). Effects of gender of reciprocal chromosomal translocation on blastocyst formation and pregnancy outcome in preimplantation genetic testing. Front Endocrinol.

[9] Boynukalin F, Gultomruk M, Turgut N, Rubio C, Rodrigo L, Yarkiner Z (2021). The impact of patient, embryo, and translocation characteristics on the ploidy status of young couples undergoing preimplantation genetic testing for structural rearrangements (PGT-SR) by next generation sequencing (NGS). J Assist Reprod Genet.

[10] Liu H, Mao B, Xu X, Liu L, Ma X, Zhang X (2020). The effectiveness of Next-Generation sequencing-based preimplantation genetic testing for balanced translocation couples. Cytogenet Genome Res.

[11] Ahdad N, Mayeur A, Hesters L, Grynberg M, Frydman N. Does the prognosis of PGT-SR differ in female translocation carriers compare to male carriers? A lesson learned from 331 couples. Reprod Biomed Online. 2020;40(5). 10.1016/j.rbmo.2020.01.025 32334941

[12] Zhang Z, Zhang L, Wang Y, Bi X, Liang L, Yuan Y (2022). Logistic regression analyses of factors affecting the euploidy of blastocysts undergoing in vitro fertilization and preimplantation genetic testing. Medicine.

[13] Yunxia L. .P.C. [Analysis on the outcome of preimplantation genetic diagnosis of assisted pregnancy in chromosome translocation carriers of different genders]. Chin Reprod Contracept. 2019 (08):622–7.

[14] Hunt PA, Hassold TJ (2002). Sex matters in meiosis. Science.

[15] Wang J, Li D, Xu Z, Diao Z, Zhou J, Lin F (2019). Analysis of meiotic segregation modes in biopsied blastocysts from preimplantation genetic testing cycles of reciprocal translocations. Mol Cytogenet.

[16] Liu M, Bu Z, Liu Y, Liu J, Dai S (2022). Are ovarian responses and the number of transferable embryos different in females and partners of male balanced translocation carriers?. J Assist Reprod Genet.

[17] Xiaojuan X, Rui Z, Wei W, Hongfang L, Lin L, Bin M et al. The effect of chromosomal polymorphisms on the outcomes of fresh IVF/ICSI-ET cycles in a Chinese population. J Assist Reprod Genet. 2016;33(11). 10.1007/s10815-016-0793-2 PMC5125150 27544276

[18] Liang J, Zhang Y, Yu Y, Sun W, Jing J, Liu R (2014). Effect of chromosomal polymorphisms of different genders on fertilization rate of fresh IVF-ICSI embryo transfer cycles. Reprod Biomed Online.

[19] Zhanhui O, Minna Y, Zhiheng C, Ling S. Meta-analysis of the association between chromosomal polymorphisms and outcomes of embryo transfer following in vitro fertilization and/or intracytoplasmic sperm injection. Int J Gynaecol Obstet. 2019;144(2). 10.1002/ijgo.12702 30378097

[20] Ogur C, Kahraman S, Griffin D, Cinar Yapan C, Tufekci M, Cetinkaya M (2023). PGT for structural chromosomal rearrangements in 300 couples reveals specific risk factors but an interchromosomal effect is unlikely. Reprod Biomed Online.

[21] Wang H. Introduction and interpretation of the updated contents of the International System for Human Cytogenomic nomenclature (ISCN 2020) Chin J Med Genet. 2021;38(12). 10.3760/cma.j.cn511374-20210304-00184 34839499

[22] Hao W. [Introduction and interpretation of the updated contents of the International System for Human Cytogenomic nomenclature]. Zhonghua Yi Xue Yi Chuan Xue Za Zhi = Zhonghua Yixue Yichuanxue zazhi = Chinese J Med Genet. 2021;38(12). 10.3760/cma.j.cn511374-20210304-00184 34839499

[23] Mateu-Brull E, Rodrigo L, Peinado V, Mercader A, Campos-Galindo I, Bronet F (2019). Interchromosomal effect in carriers of translocations and inversions assessed by preimplantation genetic testing for structural rearrangements (PGT-SR). J Assist Reprod Genet.

[24] Wu S, Zhang Y, Liu X, Liu J (2022). The relationship between the available blastocysts formation rate and chromosome balanced translocation. J Practical Med.

[25] Liu J, Kong H, Yu X, Zhou M, Liu X, Liu X (2022). In vitroThe role of endometrial thickness in predicting ectopic pregnancy after fertilization and the establishment of a prediction model. Front Endocrinol.

[26] Jia L, Chen PY, Guo YC, Zhang ZQ, Fang C (2020). Prediction of cumulative live birth rate in women aged 40 years and over undergoing in vitro fertilization/intracytoplasmic sperm injectiona. Reproductive and Developmental Medicine.

[27] The Vienna consensus (2017). Report of an expert meeting on the development of ART laboratory performance indicators. Reprod Biomed Online.

[28] Gardner D, Lane M, Stevens J, Schlenker T, Schoolcraft W (2000). Blastocyst score affects implantation and pregnancy outcome: towards a single blastocyst transfer. Fertil Steril.

[29] Findikli N, Kahraman S, Kumtepe Y, Donmez E, Biricik A, Sertyel S (2003). Embryo development characteristics in Robertsonian and reciprocal translocations: a comparison of results with non-translocation cases. Reprod Biomed Online.

[30] Zhang L, Wei D, Zhu Y, Jiang W, Xia M, Li J (2019). Interaction of acrocentric chromosome involved in translocation and sex of the carrier influences the proportion of alternate segregation in autosomal reciprocal translocations. Hum Reprod (Oxford England).

[31] Zhang L, Jiang W, Zhu Y, Chen H, Yan J, Chen Z (2019). Effects of a carrier’s sex and age on the segregation patterns of the trivalent of Robertsonian translocations. J Assist Reprod Genet.

[32] Garagna S, Zuccotti M, Thornhill A, Fernandez-Donoso R, Berrios S, Capanna E (2001). Alteration of nuclear architecture in male germ cells of chromosomally derived subfertile mice. J Cell Sci.

[33] Baccetti B, Capitani S, Collodel G, Estenoz M, Gambera L, Piomboni P. Infertile spermatozoa in a human carrier of robertsonian translocation 14;22. Fertil Steril. 2002;78(5). 10.1016/s0015-0282(02)03379-4 12414006

[34] Shuo Z, Caixia L, Junping W, Haiyan S, Jing Z, Saijuan Z et al. Analysis of segregation patterns of quadrivalent structures and the effect on genome stability during meiosis in reciprocal translocation carriers. Human reproduction (Oxford, England). 2018;33(4). 10.1093/humrep/dey036 29579270

[35] Brugnon F, Janny L, Communal Y, Darcha C, Szczepaniak C, Pellestor F (2010). Apoptosis and meiotic segregation in ejaculated sperm from robertsonian translocation carrier patients. Hum Reprod.

[36] Rouen A, Pyram K, Pollet-Villard X, Hyon C, Dorna M, Marques S (2013). Simultaneous cell by cell study of both DNA fragmentation and chromosomal segregation in spermatozoa from chromosomal rearrangement carriers. J Assist Reprod Genet.

[37] Amir H, Barbash-Hazan S, Kalma Y, Frumkin T, Malcov M, Samara N (2019). Time-lapse imaging reveals delayed development of embryos carrying unbalanced chromosomal translocations. J Assist Reprod Genet.

[38] Beyer C, Willats E (2017). Natural selection between day 3 and day 5/6 PGD embryos in couples with reciprocal or robertsonian translocations. J Assist Reprod Genet.

[39] Bo P, Julang L (2019). The art of oocyte meiotic arrest regulation.

[40] H WW. Regulation of mammalian spermatogenesis by miRNAs. Semin Cell Dev Biol. 2021;121. 10.1016/j.semcdb.2021.05.009 PMC8591147 34006455

[41] E H, S L, S S-J. The role of DNA strand breaks in human spermatozoa used for IVF and ICSI. Acta Obstet Gynecol Scand. 2000;79(7). 10929955

[42] Cassuto NG, Foll NL, Chantot-Bastaraud S, Balet R, Bouret D, Rouen A et al. Sperm fluorescence in situ hybridization study in nine men carrying a Robertsonian or a reciprocal translocation: relationship between segregation modes and high-magnification sperm morphology examination. Fertil Steril. 2011;96(4). 10.1016/j.fertnstert.2011.07.1143 21871621

[43] Perrin A, Caer E, Oliver-Bonet M, Navarro J, Benet J, Amice V et al. DNA fragmentation and meiotic segregation in sperm of carriers of a chromosomal structural abnormality. Fertil Steril. 2008;92(2). 10.1016/j.fertnstert.2008.06.052 18706548

[44] Thanassoulas A, Swann K, Lai F, Nomikos M, SPERM FACTORS, AND EGG ACTIVATION (2022). The structure and function relationship of sperm PLCZ1. Reprod (Cambridge England).

[45] Mi CE, Eun HJ, Pyung KI, Sik LW, Ki YT, Han SS. Preimplantation genetic diagnosis for couples with a robertsonian translocation: practical information for genetic counseling. J Assist Reprod Genet. 2012;29(1). 10.1007/s10815-011-9654-1 PMC3252419 22081077

[46] Stahl A, Luciani J, Hartung M, Devictor M, Bergé-Lefranc J, Guichaoua M (1983). Structural basis for Robertsonian translocations in man: association of ribosomal genes in the nucleolar fibrillar center in meiotic spermatocytes and oocytes. Proc Natl Acad Sci USA.

[47] E CP, E PS, W PJ. Genetic analysis of chromosome pairing, recombination, and cell cycle control during first meiotic prophase in mammals. Endocr Rev. 2006;27(4). 10.1210/er.2005-0017 16543383

[48] Manterola M, Page J, Vasco C, Berríos S, Parra MT, Viera A et al. A high incidence of meiotic silencing of unsynapsed chromatin is not associated with substantial pachytene loss in heterozygous male mice carrying multiple simple robertsonian translocations. PLoS Genet. 2009;5(8). 10.1371/journal.pgen.1000625 PMC2726437 19714216

[49] Cai Y, Ding M, Lin F, Diao Z, Zhang N, Sun H (2019). Evaluation of preimplantation genetic testing based on next-generation sequencing for balanced reciprocal translocation carriers. Reprod Biomed Online.

[50] Alfarawati S, Fragouli E, Colls P, Stevens J, Gutiérrez-Mateo C, Schoolcraft W (2011). The relationship between blastocyst morphology, chromosomal abnormality, and embryo gender. Fertil Steril.

[51] Charalambous C, Webster A, Schuh M (2023). Aneuploidy in mammalian oocytes and the impact of maternal ageing. Nat Rev Mol Cell Biol.

[52] Fragouli E, Katz-Jaffe M, Alfarawati S, Stevens J, Colls P, Goodall N (2010). Comprehensive chromosome screening of polar bodies and blastocysts from couples experiencing repeated implantation failure. Fertil Steril.

