# Supplementary material for: Effects of chromosomal translocation characteristics on fertilization and blastocyst development — a retrospective cohort study
Source: BMC Med Genomics. 2023 Nov 1;16:273. doi: 10.1186/s12920-023-01715-4 (PMC10619257; doi:10.1186/s12920-023-01715-4)
Supplement: Supplementary file 1 — Supplementary Material 1 [file 12920_2023_1715_MOESM1_ESM.docx]

Supplemental table 1 Univariate linear regression analysis of factors related to fertilization and blastocyst development in RecT patients

|  | fertilization rate | | blastocyst formation rate | | available blastocyst rate | |
| --- | --- | --- | --- | --- | --- | --- |
|  | *B(95%CI)* | *P* | *B(95%CI)* | *P* | *B(95%CI)* | *P* |
| Female age (years) | -0.001(-0.005, 0.003) | 0.645 | -0.003(-0.010, 0.003) | 0.302 | -0.002(-0.007, 0.004) | 0.592 |
| Male age (years) | -0.003(-0.007, 0.001) | 0.179 | -0.007(-0.013, -0.001) | 0.013^*^ | -0.007(-0.012, -0.002) | 0.009^*^ |
| Female BMI (kg/m2) | -0.002(-0.009, 0.004) | 0.471 | 0.001(-0.008, 0.010) | 0.790 | -0.003(-0.011, 0.005) | 0.494 |
| Basal FSH (U/L) | -0.006(-0.016, 0.005) | 0.277 | 0.007(-0.008, 0.021) | 0.382 | 0.003(-0.010, 0.016) | 0.646 |
| Basal LH (U/L) | 0.002(-0.003,0.007) | 0.432 | 0.001(-0.006,0.009) | 0.699 | 0.003(-0.004,0.009) | 0.448 |
| AMH（pmol/L） | 0.001(-0.001, 0.002) | 0.222 | 0.001(-0.001, 0.002) | 0.255 | 0.001(-0.001, 0.002) | 0.086 |
| Gn dose/1000 (U) | -0.033(-0.055,-0.012) | 0.003^*^ | -0.026(-0.057, 0.005) | 0.101 | -0.037(-0.065, -0.009) | 0.009^*^ |
| Days of Gn | -0.016(-0.025,-0.006) | 0.002^*^ | -0.013(-0.027, 0.001) | 0.078 | -0.016(-0.029, -0.004) | 0.011^*^ |
| Semen motility | 0.001(-0.001,0.003) | 0.150 | 0.002(-0.001,0.004) | 0.058 | 0.002(-0.001, 0.004) | 0.142 |
| Semen volume | 0.042(-0.031,0.114) | 0.263 | 0.044(-0.065,0.153) | 0.426 | 0.021(-0.077, 0.119) | 0.672 |

Notes: RecT represents Reciprocal translocation; BMI represents body mass index；AMH represents anti-Müllerian hormone; LH represents luteinizing hormone; FSH represents follicle-stimulating hormone; Gn represents gonadotropins. * represents *P*<0.05

Supplemental table 2 Univariate linear regression analysis of factors related to fertilization and blastocyst development in RobT patients

|  | fertilization rate | | blastocyst formation rate | | available blastocyst rate | |
| --- | --- | --- | --- | --- | --- | --- |
|  | *B(95%CI)* | *P* | *B(95%CI)* | *P* | *B(95%CI)* | *P* |
| Female age (years) | <-0.001(-0.009, 0.009) | 0.997 | -0.001(-0.013,0.010) | 0.846 | 0.004(-0.008, 0.015) | 0.505 |
| Male age (years) | 0.001(-0.008, 0.010) | 0.843 | 0.005(-0.007,0.016) | 0.401 | 0.008(-0.004, 0.019) | 0.178 |
| Female BMI (kg/m2) | -0.003(-0.014, 0.008) | 0.544 | -0.003(-0.016,0.011) | 0.666 | -0.008(-0.021, 0.005) | 0.212 |
| Basal FSH (U/L) | <0.001(-0.001, 0.001) | 0.789 | 0.001(-0.001,0.002) | 0.100 | 0.001(0.0001, 0.002) | 0.024^*^ |
| Basal LH (U/L) | 0.010(-0.001,0.021) | 0.084 | -0..001(-0.015,0.012) | 0.833 | -0.003(-0.017,0.010) | 0.617 |
| AMH（pmol/L） | 0.002(-0.001, 0.004) | 0.036^*^ | <0.001(-0.002,0.003) | 0.781 | <0.001(-0.003, 0.002) | 0.810 |
| Gn dose/1000 (U) | -0.015(-0.056, 0.027) | 0.490 | -0.017(-0.066,0.031) | 0.476 | 0.010(-0.037,0.058) | 0.665 |
| Days of Gn | -0.003(-0.020, 0.014) | 0.750 | -0.006(-0.026,0.015) | 0.587 | 0.012(-0.007, 0.032) | 0.209 |
| Semen motility | 0.001(-0.001,0.003) | 0.404 | -0.001(-0.004,0.002) | 0.452 | 0.001(-0.002, 0.004) | 0.420 |
| Semen volume | 0.035(-0.076,0.146) | 0.530 | 0.085(-0.051,0.220) | 0219 | 0.072(-0.060, 0.205) | 0.282 |

Notes: RobT represents Robertsonian translocation; BMI represents body mass index；AMH represents anti-Müllerian hormone；LH represents luteinizing hormone ;FSH represents follicle-stimulating hormone; Gn represents gonadotropins. * represents *P*<0.05

Supplemental table 3 Univariate linear regression analysis of factors related to fertilization and blastocyst development in female patients

|  | fertilization rate | | blastocyst formation rate | | available blastocyst rate | |
| --- | --- | --- | --- | --- | --- | --- |
|  | *B(95%CI)* | *P* | *B(95%CI)* | *P* | *B(95%CI)* | *P* |
| Female age (years) | -0.004(-0.009,0.001) | 0.120 | 0.001(-0.007,0.008) | 0.844 | 0.002(-0.005,0.009) | 0.654 |
| Male age (years) | -0.007(-0.012,-0.002) | 0.007^*^ | -0.004(-0.011,0.004) | 0.327 | -0.003(-0.010,0.004) | 0.392 |
| Female BMI (kg/m2) | <-0.001(-0.007,0.007) | 0.979 | 0.004(-0.006,0.015) | 0.439 | -0.003(-0.013,0.007) | 0.559 |
| Basal FSH (U/L) | -0.011(-0.022,0.001) | 0.084 | 0.004(-0.014, 0.021) | 0.664 | 0.004(-0.012,0.020) | 0.632 |
| Basal LH (U/L) | <0.001(-0.005,0.006) | 0.927 | -0.002(-0.010,0.006) | 0.611 | -0.002(-0.010,0.005) | 0.531 |
| AMH（pmol/l） | 0.002(0.0004, 0.003) | 0.008^*^ | 0.001(-0.001, 0.003) | 0.257 | 0.001(-0.001,0.002) | 0.478 |
| Gn dose/1000 (U) | -0.025(-0.050,-0.0002) | 0.052 | -0.025(-0.062,0.001) | 0.171 | -0.022(-0.058,0.012) | 0.201 |
| Days of Gn | -0.005(-0.017,0.007) | 0.384 | -0.013(-0.031, 0.004) | 0.130 | -0.012(-0.028,0.004) | 0.141 |
| Semen motility | 0.001(-0.001,0.003) | 0.317 | 0.002(-0.001,0.005) | 0.126 | 0.003(-0.001,0.005) | 0.078 |
| Semen volume | -0.001(-0.086, 0.084) | 0.984 | 0.017(-0.106,0.140) | 0.783 | 0.011(-0.104, 0.127) | 0.844 |

Notes: BMI represents body mass index；AMH represents anti-Müllerian hormone；FSH represents follicle-stimulating hormone；LH represents luteinizing hormone; Gn represents gonadotropins. * represents *P*<0.05

Supplemental table 4 Univariate linear regression analysis of factors related to fertilization and blastocyst development in male patients

|  | fertilization rate | | blastocyst formation rate | | available blastocyst rate | |
| --- | --- | --- | --- | --- | --- | --- |
|  | *B(95.0%CI)* | *P* | *B(95.0%CI)* | *P* | *B(95.0%CI)* | *P* |
| Female age (years) | 0.002(-0.004,0.008) | 0.443 | -0.007(-0.015,0.001) | 0.103 | -0.003(-0.010,0.005) | 0.450 |
| Male age (years) | 0.002(-0.003,0.007) | 0.482 | -0.006(-0.013,0.001) | 0.076 | -0.005(-0.012,0.001) | 0.093 |
| Female BMI (kg/m2) | -0.005(-0.013,0.003) | 0.196 | -0.004(-0.015,0.007) | 0.491 | -0.006(-0.016,0.004) | 0.255 |
| Basal FSH (U/L) | <0.001(-0.001,0.001) | 0.788 | 0.001(-0.0002,0.002) | 0.103 | 0.001(0.0003,0.002) | 0.015^*^ |
| Basal LH (U/L) | 0.008(0.0004, 0.016) | 0.040^*^ | 0.006(-0.005,0.016) | 0.312 | 0.007(-0.003,0.016) | 0.180 |
| AMH（pmol/l） | <0.001(-0.001,0.002) | 0.651 | <0.001(-0.001,0.002) | 0.625 | 0.001(-0.001,0.002) | 0.264 |
| Gn dose/1000 (U) | -0.033(-0.062,-0.004) | 0.024^*^ | -0.023(-0.061,0.015) | 0.241 | -0.029(-0.063, 0.006) | 0.106 |
| Days of Gn | -0.017(-0.029,-0.006) | 0.004^*^ | -0.008(-0.024,0.007) | 0.291 | -0.005(-0.019,0.010) | 0.522 |
| Semen motility | 0.001(-0.001,0.003) | 0.324 | <0.001(-0.002,0.003) | 0.822 | <0.001(-0.002,0.002) | 0.884 |
| Semen volume | 0.076(-0.010, 0.162) | 0.082 | 0.091(-0.029,0.211) | 0.138 | 0.054(-0.056, 0.165) | 0.332 |

Notes: BMI represents body mass index；AMH represents anti-Müllerian hormone；FSH represents follicle-stimulating hormone；LH represents luteinizing hormone; Gn represents gonadotropins； * represents *P*<0.05
